# Perceptions of caregivers about health and nutritional problems and feeding practices of infants: a qualitative study on exclusive breast-feeding in Kwale, Kenya

**DOI:** 10.1186/1471-2458-13-525

**Published:** 2013-05-30

**Authors:** Akiko Matsuyama, Mohamed Karama, Junichi Tanaka, Satoshi Kaneko

**Affiliations:** 1 School of International Health Development, Nagasaki University, Nagasaki, Japan; 2 Centre for Public Health Research (CPHR), Kenya Medical Research Institute, (KEMRI), Kenya; 3 Institute of Tropical Medicine, Nagasaki University, Nagasaki, Japan

**Keywords:** Exclusive breast-feeding, Local etiology, Insufficient breast milk, Qualitative study

## Abstract

**Background:**

Despite the significant positive effect of exclusive breast-feeding on child health, only 32% of children under 6 months old were exclusively breast-fed in Kenya in 2008. The aim of this study was to explore perceptions and feeding practices of caregivers of children under 6 months old with special attention to the caregivers’ indigenous knowledge, perceptions about the health and nutritional problems of their infants, and care-seeking behaviors that affect feeding practices.

**Methods:**

The study was exploratory and used an inductive approach. In all, 32 key informants, including mothers, mothers-in-law, and traditional healers, were interviewed in-depth. The number of participants in free-listing of perceived health problems of babies, in ranking of the perceived severity of these health problems, and in free-listing of food and drink given to children under 6 months old were 29, 28, and 32, respectively. Additionally, 28 babies under 6 months old were observed at home with regard to feeding practices. Data obtained using these methods were triangulated to formulate an ethnomedical explanatory model for mothers who do not practice exclusive breast-feeding.

**Results:**

The informants stated that various types of food, drink, and medicine were given to infants under 6 months old. Direct observation also confirmed that 2- to 3-month-old babies were given porridge, water, juice, herbal medicine, and over-the-counter medicine. Mothers’ perceptions of insufficient breast milk production and a lack of proper knowledge about the value of breast milk were identified in key informant interviews, free-listing, and ranking as important factors associating with the use of food and drink other than breast milk; in addition, perceived ill health of babies appears to be associated with suboptimal practice of exclusive breast-feeding. Caregivers used various folk and popular medicines from the drugstore, their own backyard or garden, and traditional healers so that the mother or child would not be exposed to perceived risks during the vulnerable period after birth.

**Conclusions:**

Mothers should be advised during their antenatal and postnatal care about exclusive breast-feeding. This should be done not as a single vertical message, but in relation to their concerns about the health and nutritional problems of their babies.

## Background

The effects of malnutrition on child morbidity and mortality have been widely documented [1-7]. It is estimated that maternal and child undernutrition are the underlying cause of 3.5 million deaths—35% of the disease burden in children younger than 5 years [1]. Among several child survival interventions, exclusive breast-feeding during the first 6 months of life and continued breast-feeding at least up to 12 months have been shown to have an effect on reducing mortality from the major causes of childhood death, such as hypothermia, hypoglycemia [8], diarrhea, pneumonia and neonatal sepsis [1,9]. Nevertheless, nutrition is an often-neglected aspect of maternal, newborn, and child health [10]. The underlying problems of nutrition are seen as difficult to tackle, because they are often associated with cultural issues, and no drug, vaccine, or product can change people’s behavior [11].

Kenya is listed as one of the top 20 countries in the world with the greatest level of undernutrition [12]. In 2008–09, the proportion of stunted children aged 6–59 months old was reported as 29.6%, and 20.3% were underweight [13]. Although trend data suggest that the prevalence of exclusive breast-feeding among infants younger than 6 months in 66 developing countries increased from 33% in 1995 to 39% in 2010 [14], only 31.9% of children under 6 months old were exclusively breast-fed in Kenya in 2008–09 [13]. Though some epidemiological studies have examined breast-feeding practices in Kenya [15], the complexity of feeding behavior of caregivers—particularly relating to why exclusive breast-feeding is not practiced—has not been fully explored. The aim of the present study was to explore perceptions and feeding practices used by caregivers of children under 6 months old in Kwale, Kenya. Among the caregivers, special attention was paid to their indigenous knowledge, their perceptions about the health and nutritional problems of their infants, and their care-seeking behaviors that affect feeding practices in their sociocultural context.

## Methods

### Study site

The study was conducted in Kwale in the Coast Province of southern Kenya from July to August and in December 2011. Kwale is approximately 30 km southwest of Mombasa and 15 km inland toward the Tanzanian border. The Coast Province has a relatively high child mortality rate (71 per 1,000 live births), which exceeds the national average of 52 per 1,000 live births [14]. Malaria, schistosomiasis, filariasis, HIV, and tuberculosis are endemic in the region around Kwale [16]. The majority of the population in Kwale lives on subsistence agriculture. The principal religion is Islam.

The Health and Demographic Surveillance System (HDSS) has been implemented in the area by the Kenya Medical Research Institute in collaboration with Nagasaki University. From the HDSS, vital records for the population of 42,585 (as of 1 July, 2009) [16] were available, and a part of these data was used in the present study to verify the age of the children.

### Study design

This study adopted an exploratory and inductive approach. It employed qualitative methods to identify how and why caregivers engaged in certain feeding practices. First, key informant interviews [17,18] were conducted with mothers, mothers-in-law, and husbands to acquire in-depth information relating to feeding practices, perceptions, and care-seeking behavior for the infants. We also interviewed local health practitioners, such as traditional birth attendants (TBAs) and traditional healers, to understand their perspectives on health problems of the infants and the advice and treatment given by these practitioners. The interviews were undertaken throughout the study period, and the information was used to determine the overall feeding situation; it was also used to confirm and elicit further details after data from other methods, such as free-listing, ranking, and direct observation, were obtained.

Second, more structured interview techniques, such as free-listing and ranking, were employed. The free-listing data were used for cultural domain analysis to elicit how people in a group thought about lists of things that ‘somehow go together’. A domain may be defined as an organized set of words, concepts, or sentences, all on the same level of contrast, that jointly refer to a single conceptual sphere [17]. The goal of such a data collection method was to understand how people in different cultures interpret the content of the domains differently [17,18], e.g., “perceived health problems of the infants” and “foods and drinks to be given to the infants,” for this study. Ranking was performed with the participants, based on the free-listing of perceived health problems of infants, to elicit the perceived severity of health problems [17] and care or treatment sought for each problem. Additionally, direct observation [18] was undertaken to improve the validity of key informant interview and free-listing data. This observation was conducted in the households of children under 6 months old during the daytime: the focus was on feeding practices, and it was complemented by an interview to record caregivers’ behavior from the previous evening. This resulted in 24-hour feeding practice records.

### Data collection and analysis

One of the authors undertook the key informant interviews together with a trained research assistant from the study area. For free-listing, ranking, and direct observation, we used five data collectors from the study area who were trained in the methods. We used purposive sampling for the key informant interviews and convenience sampling for the other methods [17]. After consultation with local HDSS field interviewers, we identified the caregivers of infants and younger children living within a few kilometers of two health centers in Kwale as the key informants. Sixteen mothers, seven mothers-in-law, and two husbands were interviewed. Two TBAs and four traditional healers were also included as key informants. Only one member of the health staff (a nurse at a health center) was interviewed owing to time constraints.

Using the ethnographic interview guide, we asked a wide range of open-ended questions. The questions included aspects related to the following: care of the young infants (including that related to the *arobani* taboo—see below); how breast-feeding was initiated; how long the infants were breast-fed exclusively (and how long with complementary food); the kind of food and drink given to the infants and when they started receiving them; health problems of the infants and the perceived causes; and advice and care they sought.

For free-listing, convenience sampling was adopted. With reference to the HDSS dataset, we selected 30 mothers with at least one child under 2 years old and who lived within approximately 2 km of two health centers in Kwale. The free-listing exercise involved two topics. The first topic concerned “health problems of children under 6 months old”. The mothers were asked to list all possible health problems they thought that newborns and infants might have and what they believed the causes of each health problem to be. In all, 29 mothers completed the exercise and the aggregated list of these mothers’ responses was used for the ranking exercise. Data collectors visited another set of 30 mothers, who were selected using the same criteria as the previous set. The aggregated list of the top 15 health problems most frequently cited by the free-listing participants was read out individually to the second set of mothers, who were asked to choose one of three levels of severity for each health problem; serious, intermediate, and mild. A health problem categorized as “serious” was scored as 2, “intermediate” was scored as 1, and “mild” was scored as 0. The higher the aggregated score of the health problem, the higher was the ranking and perceived severity. Of the 30 mothers, 28 completed this exercise.

The topic of the second free-listing, which was conducted about a week after the first, related to “kind of food and drink given to infants under 6 months old”. In all, 32 mothers—including some who undertook free-listing of the first topic and the ranking exercises—were individually asked to list all possible kinds of food and drink given to children. For direct observation, we selected from the HDSS dataset 30 households having at least one child under 6 months old living within approximately 2 km of the main two health centers in the area. The data collectors visited the households and observed what and how the index child was fed from 8 a.m. to 5 p.m. Then, the child’s caregiver was asked what kind of food and drink had been given to the infant the previous day from 5 p.m. to 8 a.m. to complete the 24-hour data on feeding. These observations were completed for 28 of the 30 households.

All data were recorded in the local language (Swahili) and translated into English. The information gathered using these methods was analyzed based on grounded theory [18,19]. The text of the key informant interviews was transcribed and reviewed to identify categories and concepts by means of inductive coding. Data from the direct observation with recall interviews were used to verify the foods and drinks for identification in the interviews and free-listing exercises. Data generated with these methods were triangulated [18-20], to construct an indigenous explanatory model [21,22] of the caregivers that did not practice exclusive breast-feeding. For example, perceived health problems of the infants identified by the free-listing were further detailed during the interview. Some types of food and drink for the children under 6 months old elicited through key informant interviews and free-listing were further confirmed during the direct observation.

The ethical committee of the Kenya Medical Research Institute, Kenya, and the ethical committee of the Institute for Tropical Medicine, Nagasaki University, provided approval to conduct the HDSS in Kwale, Kenya, of which this study was one component. Each participant in the study provided informed consent for participation.

## Results

### Characteristics of the study participants

The mean age of the 16 mothers who participated in the key informant interviews was 27.2 years old; that of the seven mothers-in-law was 60.6 years old. The two husbands were approximately 30 years old. The average age of the two TBAs was 58.0 years. The mean age of the participants (mothers) for the free-listing (topics 1 and 2) and the ranking was 26.2, 24.0, and 26.4 years, respectively. The mean age of mothers with a child under 6 months old for direct observation was 21.0 years old. With regard to educational level, the majority of mothers who participated in the key informant interviews, free-listing, ranking, and direct observation attended elementary school, but almost half of those did not complete their education there. With all the various methods used, the mothers had average of approximately three children (Table 1).

**Table 1:** Characteristics of the study participants

**Key informant interview**						
	**Mother (n = 16)**	**Mother -in-law (n = 9)**	**Husband (n = 2)**	**Traditional birth attendant (n = 2)**	**Traditional healer (n = 4)**	**Health staff (n = 1)**
**Age** (mean)	27.2 years	60.6 years	30.5 years	58.0 years	43.5 years	43.0 years
**Education**						
No education	3 (8.8%)	8	0	1	1	0
Primary incomplete	7 (43.8%)	1	0	1	2	0
Primary complete	5 (31.3%)	0	1	0	1	0
Secondary +	1 (6.3%)	0	1	0	0	1
**No. of living children** (mean)	3.4	N.A.	3.0	N.A.	N.A.	N.A.
**Free listing (Topic 1)** Mother (n = 29)						
**Age** (mean)	26.2 years	N.A				
**Education**						
No education	6 (20.7%)					
Primary incomplete	10 (34.5%)					
Primary complete	12 (41.4%)					
Secondary +	1 (3.4%)					
**No. of living children** (mean)	3.2					
**Free listing (Topic 2)** Mother (n = 28)						
**Age** (mean)	24.0 years	N.A.				
**Education**						
No education	5 (17.9%)					
Primary incomplete	11 (39.3%)					
Primary complete	10(35.7%)					
Secondary +	2 (7.1%)					
**No. of living children** (mean)	3.0					
**Ranking** Mother (n = 32)						
**Age** (mean)	26.4 years	N.A.				
**Education**						
No education	3 (9.4%)					
Primary incomplete	13 (40.6%)					
Primary complete	14 (43.8%)					
Secondary +	2 (6.3%)					
**No. of living children** (mean)	3.2					
**Direct observation** Mother (n = 28)						
**Age** (mean)	21.0 years	N.A.				
**Education**						
No education	4 (14.3%)					
Primary incomplete	9 (32.1%)					
Primary complete	12 (42.9%)					
Secondary +	3 (10.7%)					
**No. of living children** (mean)	2.8					

### “Vulnerable period” for young babies

After childbirth, mothers observe a period of “pollution” for 40 days that is called *arobani* (“40 days” in Swahili). There are some taboos and practices during this period, including segregation from other family members, particularly males. The mothers are prohibited from having sexual intercourse with their husbands. Although the newborn babies are not in a state of pollution, the fact that they are physically secluded with their mothers in a corner or in another room of the house makes them inaccessible during *arobani*. Another reason for the newborns to be secluded with their mothers is the infants’ perceived vulnerability. They are “vulnerable” and prone to attack by the evil eye, an evil spirit, black magic, or “coldness”. For example, *dege*, a kind of evil eye, attacks when a baby happens to drink breast milk at the same time as an outsider sees the baby and swallows his or her own saliva; the baby will then develop a swollen stomach, stomachache, or diarrhea. *Dzongo*, another type of evil eye, is produced through envy or ill intentions by people who falsely praise the baby as being “healthy”, “beautiful”, or “smart”; this makes the baby ill through stomachache and diarrhea. Infants may also be caught by an evil eye when they or their mothers are seen by outsiders during meals or breast-feeding. Diarrhea is also believed to occur if an eagle sees a baby as it flies over the village; this may lead to more ill health, such as *nyuni*, which is characterized by fits and rolling eyes. Extramarital relationships by the father during *arobani* are believed to be another cause of ill health in the newborns: an amulet, *mapande*, given by the traditional healer is wrapped around the baby’s waist to prevent it from falling ill through the father’s misconduct. Moreover, the newborns are supposed to be prone to the above-mentioned coldness, which typically enters through the fontanels or toes. Wrapping up the whole body of a newborn with a large piece of local cloth, *tinga*, or putting woolen caps or socks onto the baby is done to protect the child from the perceived risks of the outside world during this vulnerable period.

Many key informants reported that the babies were mostly breast-fed for the first 5–6 months. However, they added that other food and drink were also given if the mother’s milk was insufficient. Some women were actually witnessed giving their babies some food or drink by means of a syringe, a bottle, or their fingers during the home-visit interviews. Moreover, the key informants often spoke of homemade herbal or over-the-counter medicines being given, particularly for the common illnesses of small babies. In the area, there were also some traditional healers known as *mganga wa nyuni* (“doctor for baby fits”), who specialized in childhood illnesses, even though general traditional healers treat various type of health problem regardless of age; such general traditional healers also handle social problems, such as divorce or business matters. *Mganga wa nyuni* claimed that the babies’ caregivers sought consultation and treatment from them for different types of health problems, including stomachache due to evil eye, bad coughs, fever, and fits. The *mganga wa nyuni* prepared herbal medicines for the caregivers or provided instruction on the making of herbal remedies at home.

### Local etiology for feeding a baby other than breast milk

#### Insufficient breast milk

A mother’s perception of insufficient breast milk is a common justification for feeding their babies *uji* (maize-based porridge), cow’s milk, *ugali* (staple food made of maize), banana, potato, bean soup, or drinks including water, tea, and orange juice.

I don’t eat well because we’re poor, and I can’t produce enough milk. That’s why my baby cries—even after breast-feeding. (Mother, 23 years old)

One young woman had given birth to a baby about a week before the interview. She stated her mother-in-law had advised her to give the baby a homemade herbal liquid since her breast milk was insufficient. Asked how she knew her daughter-in-law did not have sufficient milk, the mother-in-law appeared convinced:

The baby continues crying even after being breast-fed. It’s a peculiar way of crying: her arms and legs are stretched out. (Mother-in-law, 65 years old)

Some informants said that juice and sugar water could be used as a substitute for breast milk for newborns.

Not producing enough breast milk is quite common after delivery. A mother can feed her child juice or sugar water until she starts producing enough milk herself. (Mother, 24 years old)

#### A child needs more than breast milk to grow big and healthy

Some caregivers seemed to believe that food and drink in addition to breast milk would help their babies grow bigger or healthier. One husband explained why his baby was given drinks or foods in addition to breast milk before 6 months:

My wife thinks our baby will grow big and healthy if he’s given more food and drink, like orange juice, other than breast milk. A mixture of water, sugar, and salt also increases his appetite. (Father, 31 years old)

#### Remedies for perceived common illnesses of newborns

Many informants, including both mothers and mothers-in-law, listed cold, stomach-ache, and colic as typical health problems during the neonatal period.

Newly born babies often have a running nose because of cold, or they have stomachache or colic because of incomplete healing of the umbilical cord, unfamiliarity with breast milk, or worms in the stomach. If a baby suffers from stomachache or colic, we give them gripe water from the drugstore. If we can’t afford that, we just give them a mixture of boiled water, sugar, and salt. (Mother, 24 years old)

The health problems of newly born babies include stomachache and colds. Stomachache is caused by incomplete healing of the umbilical cord or worms in the stomach. We recognize stomachache or colic because the baby continues crying, stretching out both its arms and legs, and it doesn’t take breast milk. We then give the gripe water or a herbal medicine made from the root of *mtsalafu*. During *arobani*, small babies should not be taken outside the home because they can suffer from coldness and become ill. If they do suffer from coldness, we prepare herbal medicine and give it to them. (Mother-in-law, 55 years)

Table 2 lists the perceived common illnesses of newborns, particularly during *arobani* and the following months. The higher in the list an item appears, the more widely was it recognized as a health problem by the informants. Mothers frequently cited the following: abdominal colic, stomachache, bad cold (literally translated as “flu”), and related problems, such as coughing, vomiting, fever, diarrhea, and swollen stomach. Sore eyes or sore ears were also recognized as common problems. A state of continuous crying, *kurira*, was mentioned by some informants in the interviews, and it appeared 14^th^ in the list. Some participants said that *kurira* could be distinguished from ordinary crying by the peculiar way of both arms and legs being stretched out and that the crying often continued even after breast-feeding. It was perceived to be a sign of hunger or thirst caused by insufficient breast milk or the result of the newborn being seen by an eagle or an evil spirit. There were many perceived causes of stomachache, abdominal colic, and swollen stomach: stomachache and abdominal colic in neonates were often said to take place as a natural process during the healing of the umbilical cord; they may have been caused by a baby that was not used to breast milk, worms in the stomach, or the evil eye, including *dzongo.* A bad cold, chest cold, coughing, and fever were often associated with the above-mentioned coldness entering the child through its fontanels or toes. Mothers carrying heavy loads during pregnancy was also regarded as a cause of coughing. Suspected causes for *kuharisha* (a general term for diarrhea) were many, e.g., a mismatch of different types of foods, fever, erupting milk teeth, the child being seen by an eagle, a husband having an extramarital relationship, *dzongo*, and *nyuni* (fits, typically in children).

**Table 2:** Free-listing of perceived common illnesses in newborns and young children

**Health problem**	**# mentioned (N = 29)**	**Equivalent english term**	**Perceived causes**
1. *Ndani ya chitovu*	23	Abdominal colic	Occurs in a process of healing an umbilical cord, not used to breast milk, worm in the stomach
2. *Mafuwa*	21	“Flue (bad cold)”, Chest cold	Coldness
3. *Kukohola*	20	Coughing	Coldness, pregnant mother carrying heavy load
4. *Kuhaphika*	20	Vomiting	Fever, excessive breast-milk, *Nyuni*
5. Mwilimoto/homa	20	Fever, High temperature, “*homa*” could be malaria	Coldness, bad (cold) weather
6. *Kuharisha*	18	Diarrhea	Mismatching of different types of foods, fever, erupting tooth, eye contact with eagle, caused by stomachache, husband having an affair with other women, *Nyuni*
7. *Kulumwa ni masto*	13	Sore eye	Dust, outbreak
8. *Kulumwa ni masikiro*	9	Sore ear	Drips of water in the ears during bathing
9. *Kuodzala ndani* (*Dzongo*)	8	Stomachache, Swelling of stomachache	*Dzongo* (evil eye), baby swallowing breast-milk simultaneously the person who sees the baby swallowing ones saliva
10. *Uphere*	8	rash	Dirty clothes, dirty environment
11. *Malaria*	7	malaria	Mosquito, not using bed-net, coldness
12. *Marenjerenje/ Majipu*	7	Blister, Boil	Warm/hot weather
13. *Mwalidago*	6	Ringworm	Do not know
14. *Kurira*	6	Continuous crying	Being hungry, eye contact with eagle, evil spirit
15. *Nyuni*	6	Fits, Convulsion, Eye-ball rolling over	Eye contact with eagle, evil spirit, malaria, inheritance, scent of young leaves & shoot
16. *Pumu*	5	Chronic respiratory disease (asthma)	Coldness during rainy season
17. *Kilimi*	5	Uvula, sever coughing and vomiting	Uvula, do not know
18. *Manjano*	5	Yellow fever	Lack of blood
19. *Kukosa choo*	5	Constipation	Lack of drinking fluid and water
20. *Harara*	5	Heat rash	Warm/hot weather
21. *Mshipa* (*kwa wavulana*)	4	Abdominal pain for boys (reproductive organ problem)	Do not know, coldness
22. *Tetemaji*	3	Chicken pox	Do not know
23. *Kubanwa na mbavu*	3	Difficulties in breathing	Coldness, Nyuni, eye contact with eagle
24. *Vidonda mdomoni*	2	Sore mouth	coldness
25. *Ukambl*	2	Measles	Outbreak
26. *Kichwa*	2	Headache	A lot of stress
27. *Kifua kuma*	2	Chest pain	Drinking dirty amniotic fluid at birth
28. *Riaka*	2	Stiff neck	Do not know
29. *Kukosa ham ya kula*	1	Loss of appetite	*Dege* (baby swallowing breast-milk simultaneously the person who sees the baby swallowing ones saliva)
30. *Kifua kikuu*	1	Tuberculosis	Inheritance
31. *Kulegea*	1	Paralysis (physical disability)	Coldness
32. *Kichocho*	1	Schistosomiasis	Dirty water
33. *Kukosa Kukojia*	1	Urination problem	Do not know
34. *Kufura Miguu*	1	Swelling of legs	Do not know
35. *jasho, kutoka*	1	Sweating	Dehydration
36. *Kucha kuuma*	1	Pain of nails	Infected blood
37. *Chirwa*	1	Sickness mainly diarrhea	Father having an affair with other women
38. *Funza*	1	Jigger (chigger)	Infected blood
39. *Mwalihosi*	1	Moving of fontanel	Natural to happen to newborn

The results of ranking based on the 15 most widely recognized common health problems for small babies during *arobani* are presented in Table 3. Fits, *nyuni*, were regarded as the most serious health problem for small babies, although *nyuni* did not emerge as often as stomachache, diarrhea, fever, or colds when free-listed (Table 2). A *muganga wa nyuni*, a traditional healer specialized in child health problems stated on *nyuni* as below.

Infants that suffer from *nyuni* are brought to me. The babies get it if their mothers are possessed by evil spirits, if the babies are seen by an eagle, or if they hear the call of an eagle. Those that have *kivaduro* (serious coughing) through cold weather are given herbal medicine. If the babies become ill because of *dzongo*, it is not such a serious problem, and herbal medicine can be used to cure it. *Nyuni* particularly occurs among newly born children, and the typical symptoms are rolling eyes and fits. If the babies were taken to hospital and given chloroquine or an injection, they would die. They should be treated by traditional healers. We perform a ritual by sacrificing chickens and give them herbal medicines. (Male *mganga wa nyuni*, 53 years old).

**Table 3:** Ranking of perceived severity of health problems

	**Perceived health problem**	**Serious**	**Intermediate**	**Mild**	**Total score of 32 informants**
		**(Score = 2)**	**(Score = 1)**	**(Score = 0)**	
1	Fits, Convulsion, Eyes rolling over (*Nyuni*)	2 × **19** = 38	1 × **8** = 8	0 × **5** = 0	46
2	malaria	2 × **18** = 36	1 × **7** = 7	0 × **7** = 0	43
3	Fever, High temperature,	2 × **7** = 14	1 × **20** = 20	0 × **5** = 0	34
	“*homa*” could be malaria				
4	Ringworm	2 × **12** = 24	1 × **8** = 8	0 × **12** = 0	32
5	Continuous crying	2 × **8** = 16	1 × **15** = 15	0 × **9** = 0	31
6	Diarrhea	2 × **8** = 16	1 × **12** = 12	0 × **12** = 0	28
7	Coughing	2 × **6** = 12	1 × **15** = 15	0 × **11** = 0	27
8	Flue (bad cold)	2 × **4** = 8	1 × **18** = 18	0 × **10** = 0	26
9	Stomachache	2 × **5** = 10	1 × **12** = 12	0 × **15** = 0	22
10	Sore ear	2 × **7** = 14	1 × **6** = 6	0 × **19** = 0	20
10	Rash	2 × **2** = 4	1 × **16** = 16	0 × **14** = 0	20
11	Sore eye	2 × **3** = 6	1 × **12** = 12	0 × **17** = 0	18
12	vomiting	2 × **3** = 6	1 × 9 = 9	0 × **20** = 0	15
14	Stomachache (*dzongo*)	2 × **5** = 10	1 × **5** = 5	0 × **22** = 0	15
15	Blister	2 × **2** = 4	1 × **7** = 7	0 × **23** = 0	11

Number in bold is the number of informants classified the health problem into the level of severity.

The second-most serious health problem reported by caregivers was malaria; the third was fever, *homa*, a term that is often interchangeable with malaria. *Kurira*, continuous crying, was ranked the fifth-most serious health problem; it was higher than diarrhea, coughing, flu (bad cold), and stomachache.

Table 4 shows the top nine commonly recognized health problems from the free-listing exercise, as well as the various types of popular and folk remedies used by caregivers for each problem. Herbal medicine, such as *mzaraf* for stomachache, was typically used at home or given by a traditional healer. Many over-the-counter medicines were used in the community. Gripe water, Bonnisan, and Dentinox were very popular medicines for stomachache and were readily available at drugstores or small general stores in towns and villages. They were used for small babies with abdominal colic, which was believed to occur frequently after breast-feeding. In addition to herbal medicines for colds, fever, and coughing, Piriton Syrup, Septrin, and Panadol Syrup were commonly used over-the-counter pharmaceuticals.

**Table 4:** Folk and popular medicines for commonly recognized illnesses (the top nine problems from the free-listing exercise)

**Health problem**	**Equivalent english term**	**Perceived causes**	**Traditional care or popular medicine**
1. *Ndani ya chitovu*	Stomachache	occurs in a process of healing of umbilical cord, constipation, diarrhea	Mihaso (*Mzaraf)*, “Gripe water”, “Bonnisan”, “Dentinox”
2. *Mafuwa*	“Flue (bad cold)”, Chest cold	Coldness	“Piriton syrup”, “Septrin”, “Panadol syrup”
3. *Kukohola*	Coughing	coldness, pregnant mother carrying heavy load	Herbs, “Piriton syrup”, “Septrin”, “Pandol syrup” for vomiting perceived to be accompanied by fever
4. *Kuhaphika*	Vomiting	fever, excessive breast-milk	“Piriton syrup”, “Septrin”, “Pandol syrup” for vomiting perceived by fever
*5. Mwilimoto/homa*	Fever, High temperature, “homa” could be malaria	Coldness	“Piriton syrup”, “Septrin”, “Panadol syrup”
6. *Kuharisha*	Diarrhea	Mismatching of different types of foods, fever, erupting tooth, eye contact with eagle, husband having an affair with other women	Amulet around the baby’s waist by traditional healer (prevention), herb and salt, traditional healer, home mad ORS
7. *Kulumwa ni masto*	Sore eye	dust, outbreak	Herbs, wipe with warm water + salt, sea water, drop of breast-milk, tea leaves
8. *Kulumwa ni masikiro*	Sore ear	Drips of water in the ears during bath	Herb, coconut oil, Gun’s oil, Sheep’s oil, chicken oil
9. *Kuodzala ndani* (*Dzongo*)	Stomachache, Swelling of stomachache	*Dzongo* (evil eye), baby swallowing breast-milk simultaneously the person who sees the baby swallowing ones saliva	Prevent a baby from being seen or commented nice things by the others, traditional healer, color between eyebrows

One mother explained that she went to the health center or hospital for certain health problems, whereas traditional remedies—either from traditional healers or their own backyard or garden—were used for such illnesses as those caused by an evil eye, evil spirit, the child being seen by an eagle flying over the house, and a husband’s extramarital relationship.

There are certain health problems of small babies that can be treated only by the traditional healer, not by hospital medicine. (Mother, 22 years old)

### Food, drink, and medicine given to children under 6 months and feeding style

The free-listing exercise was performed for different types of food and drink given to children less than 6 months old. In all, 33 different types of food and drink were identified (Table 5). The item most frequently mentioned by informants was maize-based porridge, *uji*. Various types of herbs decocted for treating stomachache and abdominal colic, including *mzaraf,* were also popular. Nine types of drink and medicine in the list (underlined in Table 5) included herbal medicines prepared by traditional healers or at home in addition to over-the-counter medicines for stomachache or fever.

**Table 5:** Free-listing of the types of food, drink, and medicine given to infants under 6 months old

**Food and drink**	**# mentioned**	**Local term**	**Information**
	**(N = 28)**		
1. Maize-based porridge	27	*Uji*	Maize, water, and sometimes sugar or cow’s milk
2. Herbs/ roots	23	*Mihaso*	Including *mzaraf* for abdominal colic, stomachache
3. Cow’s milk	15	*Maziya*	
3. Ugali	15	*Sima*	
4. Banana	14	*Ndizi*	Could be mixed with mango or potato
5. Water	12	*Madzi*	
6. Potato	11	*Viazi*	
6. Bean’s soup	11	*Maharabuie mtsuzi*	
7. Medicine (not specified)	8		Mostly from hospital or over-the-counter medicine
8. Tea	7	*Chai*	
8. Orange juice	7		Often artificially sweetened juice from shop
9. Green leafy vegetables	6	*Mtsunga*	
9. Piriton syrup	6	Piriton	Over-the –counter-medicine for fever
10. Fish soup	5	*Supu ya samaki*	
11.Septrin	4	Septrin	Over-the-counter medicine for fever
11. Water + Sugar + Salt (home-made ORS?)	4		Some say to increase baby’s appetite or for thirst
11. Avocado	4	*Ovacado*	Could be mixed with milk
12. “Gripe Water”	3	Gripe water	Over-the-counter medicine for abdominal colic, stomachache
12. Bonnissan	3	Bonissan	Over-the-counter medicine for abdominal colic, stomachache
12. Passion juice	3		Often artificially sweetened juice from shop
12. Mango	3	*Embe*	
12. Rice-based porridge	3	*Mchele Mphunga*	
12. Honey	3	*Asali*	
12. Chicken soup	3		
13. Goat’s milk	2		
13. Malaria tablet	2		From hospital or over-the-counter medicine
13. Juice (not specified)	2		
14. Pancake	1	*Chapati*	
14. Biscuit	1		
14. Panadol syrup	1		Over-the-counter medicine for fever
14. Dentinox	1		Over-the-counter medicine for abdominal colic, stomachache
14. Pineapple juice	1		
14. Powder milk	1		

The results of the observation and recall interviews demonstrated that 25 of 28 caregivers had given some food, drink, or medicine to their babies under 6 months old in the 24-hour period prior to or during the observation (Table 6). Some started to give porridge, water, and juice to the newborns as early as 2–3 months old. In addition, herbal and over-the-counter medicines were given. Spoons and cups were the common feeding utensils used, but finger-feeding by caregivers, bottles of formula milk with artificial nipples, and syringes obtained at the local market were also witnessed by researchers.

**Table 6:** Kinds of food, drink, and medicine given to infants under 6 months (Total 24-hour consumption of combined direct observation and interviews)

**No**	**Age of the child**	**Kinds of food and drink given other than breast milk (All babies observed were breastfed)**	**How it is fed**	**Who feeds the child**
1	2 months	Breast milk only		Mother
2	2 months	Porridge, water	Bottle	Mother
3	3 months	Breast milk only		Mother
4	3 months	Breast-milk only	-	Mother
5	3months	Porridge	Cup	Mother
6	3 months	Porridge, water	Cup	Mother
7	3 months	“Dentanox”(medicine for stomachache/ colic), herbs	Syringe	Mother
8	4 months	Porridge	Spoon	Mother
9	4 months	Porridge	Cup	Mother
10	4 months	Porridge	Cup	Mother
11	4 months	Porridge	Spoon, cup	Mother
12	4 months	Porridge	Cup	Mother
13	4 months	Porridge	Cup	Mother
14	4 months	Porridge, water	Cup	Mother
15	4 months	Porridge, cow’s milk, water	Spoon, cup	Mother
16	4 months	Porridge, water	Spoon, cup	Mother
17	4 months	Decocted herbs	Spoon	Mother
18	4 months	Decocted herbs, “Septrin” (medicine for fever)	Spoon	Mother
19	4 months	Porridge, water	Spoon, cup	Mother
20	5 months	Porridge	Spoon, cup	Mother
21	5 months	Porridge	Bottle	Mother
22	5 months	Porridge, water, orange juice	Spoon, cup	Mother
23	5 months	Porridge, water	Spoon	Mother
24	5 months	Herbs, porridge	Spoon	Mother
25	5 months	Porridge, water	Spoon, finger	Mother
26	5 months	Porridge, water	Cup	Mother
27	5 months	Porridge, water	Spoon, cup	Mother
28	5 months	Porridge, water	Spoon	Mother

## Discussion

**Figure 1:**
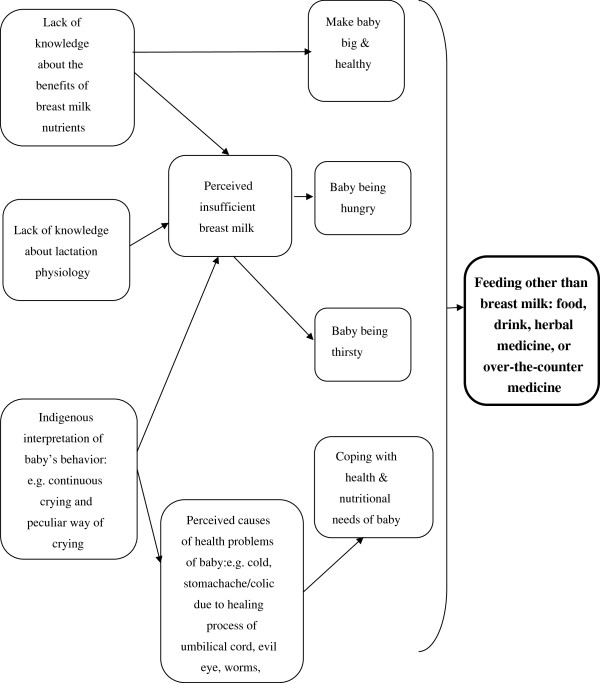
Ethnomedical model for mothers who do not practice exclusive breast-feeding.

Based on the information elicited from the interviews and free-listing, an ethnomedical explanatory model for mothers who did not practice exclusive breast-feeding was developed (Figure 1). Perceived insufficient breast milk by mothers was identified as one of the reasons. This is consistent with the findings of previous studies [23-31]. Although many informants stated that babies should be given only breast milk for the first 5–6 months of life, lack of conviction about the benefits of exclusive breast-feeding led to steps being taken to compensate for a mother’s perceived lack of breast milk. The ranking exercise also shows that *kurira*, continuous crying due to insufficient milk, was also regarded as a relatively serious health problem of babies by the mothers. Insufficient milk syndrome stems from poverty, sexism, and powerlessness, not from poor maternal nutritional status [28]. Though mothers may claim that it is their poor nutritional status, evidence suggests that maternal nutritional status has little effect on breast milk production or composition, except in severely malnourished women, i.e., those under 85% of reference weight-for-height [28]. Greiner, Esterik, and Latham argue that “it is largely a cultural phenomenon when it [insufficient milk syndrome] occurs during exclusive breast-feeding, and largely a physiological response to reduced nipple stimulation once supplemental feeding has begun” [24]. Other authors suggest that perceived breast milk inadequacy is underpinned by a complex and synergistic interaction among socio- cultural influences, feeding management such as positioning of babies and attachment while breastfeeding [32], the baby’s behavior, lactation physiology, and the mother’s psychological state [26]. Apart from perceived hunger or thirst, the observed notions of “the baby needs water” and “the baby has other needs apart from milk to grow well and faster” are also supported in the literature [29]. Furthermore, it may be the perceived ill health of the babies that makes caregivers use a variety of food and drink, other than breast milk, for feeding the infants. As shown in Table 2, stomachache, abdominal colic, diarrhea, and fever were among the most frequently recognized health problems in small babies. We have also reported (Table 4) a list of popular and folk medicines available at home and community levels. The over-the-counter medicines and herbal medicines prepared by caregivers or given by traditional healers were infused or mixed with water and given to the babies using utensils, including bottles with an artificial nipple. In the extended family, advice from significant others, such as from a mother-in-law, is influential, and such individuals often reinforce traditional norms relating to feeding practices. This observation is supported by other studies [28].

The ethnomedical explanatory model underlines the mothers’ perception of insufficient breast milk and lack of knowledge about the physiology of lactation, the benefits of breast milk, and the nutritional needs and behavior of their infants; in addition, this model accounts for their response to the perceived ill health of their babies. Mothers are often concerned about nutritional and health problems of their babies and take action in ways that are at odds with biomedicine. Scanvenius argues that “positive intention” without realizing the “negative processual consequences” most likely ends in a cessation of breast-feeding [33]. Perceived insufficient breast milk and actions taken by mothers to cope with perceived health problems in their babies may trigger a vicious circle, causing reduced production of breast milk. Infants who are given supplements in their early months spend less time breast-feeding and suckle less intensively. As a result, their mother’s breasts are less stimulated to produce milk [28,33].

More importantly, different types of folk and popular medicines can be obtained from the drugstore, own backyard or garden, or traditional healers to avoid the mother or baby being exposed to perceived risks in the outside world during the vulnerable period of *arobani* and the months that follow*.* However, the feeding styles, e.g., the caregiver using their own finger, bottles of formula milk, or syringes, may pose potential health risks to the infants.

### Limitations

There are several limitations to this study. First, it is a qualitative study, and only a small sample was included. This restricts, therefore, the generalizability of the findings. Nevertheless, the study site has relatively high child mortality and morbidity, and some of the findings on mothers’ perceptions of the health and illness of their children and their practices related to breast-feeding may be applicable to other parts of the country or other regions in a similar situation.

Second, the qualitative nature of the study raises caution with regard to inferring a causal relationship between the mothers’ perceptions as determinants and the low level of exclusive breast-feeding. Rather, insights into the mothers’ perceptions and behavior provide better understanding of the sociocultural context by which the recipients understand the exclusive breast-feeding message.

Third, this study did not investigate breast-feeding techniques, including the frequency and positions adopted by the mother and baby. It has been demonstrated that proper breast-feeding techniques can prevent, for example, nipple pain, which often leads to decreased suckling and reduced milk production [32,34,35]. If information relating to how the mothers breast-fed their children had been gathered, that would have added a very interesting dimension to the study.

Fourth, the majority of the population in Kwale is Muslim, and cultural and social norms and practices there are different from other parts of the country, where many people are Christians. It is, however, also true that regardless of religion, indigenous African culture is prevalent throughout the country. Thus, some of these findings may also be relevant to other regions.

Fifth, perceptions and practices of health professionals were not explored in this study. Previous studies have indicated that health professionals, particularly front-line workers who are in close contact with caregivers, often hold similar perceptions and attitudes to the caregivers themselves with regard to breast-feeding [36]. Consequently, health professionals fail to convince caregivers that they should alter their behavior with respect to exclusive breast-feeding. Further studies on health professionals’ knowledge, beliefs, and behavior will yield greater understanding of this issue.

Despite the limitations, this study affords qualitative information on the perceptions of mothers on their feeding practices with young children. It thus provides support for programs that aim to promote exclusive breast-feeding in the region.

## Conclusions

The present strategy of promoting exclusive breast-feeding appears to be based on the major presupposition that caregivers simply do not understand the value of breast milk. Although it appears to be the case that a lack of awareness of its advantages is one of the major reasons for failure to adopt excusive breast-feeding, additional factors may also prevent caregivers from implementing this practice. Their own strategies for coping with what they perceive as their baby’s ill health and nutritional disadvantages also seem to be related to feeding practices. When mothers are concerned about their babies’ stomachache, abdominal colic, fever, swollen stomach, or diarrhea, preaching to them out of their cultural context about the nutritional, immunological, and psychological benefits of breast-feeding may not be well received. Health professionals and community volunteers are encouraged to consult mothers during antenatal and postnatal care about exclusive breast-feeding [37]—not as a single vertical message, but in relation to their concerns about the health and nutritional problems of their babies.

## Competing interests

The authors declare that they have no competing interests.

## Authors’ contributions

AM conceived of and designed the study, contributed to the data collection, and wrote the draft of the paper. MK supervised and contributed technical support to the overall study. JT and SK contributed to designing, managing, and supervising the HDSS and gave technical and supervisory support to the study. All authors read and approved the final manuscript.

## Pre-publication history

The pre-publication history for this paper can be accessed here:

http://www.biomedcentral.com/1471-2458/13/525/prepub
